# PSSR2: a user-friendly Python package for democratizing deep learning-based point-scanning super-resolution microscopy

**DOI:** 10.1186/s44330-024-00020-5

**Published:** 2025-01-02

**Authors:** Hayden C. Stites, Uri Manor

**Affiliations:** 1 Department of Cell & Developmental Biology, School of Biological Sciences, University of California, San Diego 92093, CA, USA; 2 Halıcıoğlu Data Science Institute, University of California, San Diego 92093, CA, USA

## Abstract

**Background:**

To address the limitations of large-scale high quality microscopy image acquisition, PSSR (Point-Scanning Super-Resolution) was introduced to enhance easily acquired low quality microscopy data to a higher quality using deep learning-based methods. However, while PSSR was released as open-source, it was difficult for users to implement into their workflows due to an outdated codebase, limiting its usage by prospective users. Additionally, while the data enhancements provided by PSSR were significant, there was still potential for further improvement.

**Methods:**

To overcome this, we introduce PSSR2, a redesigned implementation of PSSR workflows and methods built to put state-of-the-art technology into the hands of the general microscopy and biology research community. PSSR2 enables user-friendly implementation of super-resolution workflows for simultaneous super-resolution and denoising of undersampled microscopy data, especially through its integrated Command Line Interface and Napari plugin. PSSR2 improves and expands upon previously established PSSR algorithms, mainly through improvements in the semi-synthetic data generation (“crappification”) and training processes.

**Results:**

In benchmarking PSSR2 on a test dataset of paired high and low resolution electron microscopy images, PSSR2 super-resolves high-resolution images from low-resolution images to a significantly higher accuracy than PSSR. The super-resolved images are also more visually representative of real-world high-resolution images.

**Discussion:**

The improvements in PSSR2, in providing higher quality images, should improve the performance of downstream analyses. We note that for accurate super-resolution, PSSR2 models should only be applied to super-resolve data sufficiently similar to training data and should be validated against real-world ground truth data.

## Introduction

The acquisition of high-quality microscopy data is time consuming and imperfect. Improvements to one of image resolution, SNR (Signal to Noise Ratio), imaging speed, or sample preservation cannot come without cost to another, requiring difficult and undesirable tradeoffs for imaging experiments. To mitigate this effect, PSSR (Point-Scanning Super-Resolution) was introduced to effectively optimize these parameters to otherwise unattainable quality [1]. Using deep learning-based super-resolution, low quality images (low resolution, low SNR) could be restored to higher quality (high resolution, high SNR). Additionally, the use of a “crappifier” to semi-synthetically degrade high quality images to their low quality counterparts via downsampling and noise injection removed the requirement of manually acquiring a training dataset comprising perfectly aligned images. A super-resolution neural network (i.e. any neural network architecture capable of predicting images of higher resolution) would then be trained by providing it with the low quality images generated via crappifier and tasking it with the prediction/super-resolution of the ground truth high quality images as training target. The original PSSR model utilized a Residual UNet (ResNet34) neural network architecture. Features are extracted via encoder blocks which progressively downscale the image followed by decoder blocks which progressively upscale the image, where respective encoder-decoder blocks are connected via skip connections. The model was trained with a mean squared error (L2) loss function, which produced excellent results validated by blinded analysis of relevant biological structures. This included the identification of presynaptic vesicles that are typically challenging, if not impossible, to accurately detect at 8nm pixel size, but were much more clearly visible in the PSSR super-resolved images at 2nm pixel size. As many large-scale connectomics EM imaging datasets are collected at 8nm pixel size in order to accelerate data acquisition, the ability to super-resolve 8nm to 2nm pixel sizes enables analysis of synaptic organelles that would otherwise be impossible.

However, the original version of PSSR was limited in implementation. For example, it only allowed for super-resolution of simple single-frame and multi-frame videomicroscopy timelapse data, not accounting for the usage of more complex multidimensional data where the benefits of PSSR may be even greater. The crappifiers used to generate training data were also imperfect, potentially contributing to decreased model accuracy when applied to real-world data.

Additionally, PSSR was also not designed with the bulk of the biological and imaging research community in mind. As machine learning becomes more widely established, the accessibility of related software becomes even more crucial. An experimental microscopist does not necessarily have experience in machine learning and/or any given programming language, and may not be able to fully benefit from, if at all, a workflow they cannot easily utilize. Underscoring this, the codebase was not structured as software, rather as a collection of undocumented functions that are not sufficient for customized usage without additional implementation. Additionally, the original code no longer runs on modern versions of the packages it is built on, which has caused difficulty for many prospective users. Thus, even though PSSR was released as open-source, it failed to maximize democratization.

To extend the democratization of PSSR, we introduce PSSR2, a Python package featuring a customizable framework for the creation of deep learning-based super-resolution/denoising imaging workflows on a wide variety of microscopy data including both light and electron microscopy. To this end, we redesigned the PSSR approach from the ground up with ease of use and wide applicability in highest priority, while also addressing the longstanding issues facing the original implementation. Additionally, while the main objective of PSSR2 is to provide an accessible yet fully-featured package for practical microscopy super-resolution, PSSR2 also improves on existing PSSR algorithms to allow for more accurate model training and subsequent model predictions.

The usage of the Python programming language allows for a user-friendly modular package-based interface and integration with widely used research software, such as PyTorch [2] and Napari [3]. PSSR2 is accessible through multiple entry points of varying complexities and required technical knowledge, while all including the same core features. For advanced users, lower level features are exposed in a way that allows for straightforward modification of existing processes and addition of custom logic. PSSR2 also boasts newly trained models with state-of-the-art performance on real-world data, substantially improving on prior PSSR implementations and other competing approaches.

## Methods

### Application features

In order to produce a straightforward yet widely applicable PSSR2, we completely rebuilt PSSR from the ground up. In doing so, we simplify and improve important elements of the PSSR workflow while simultaneously allowing PSSR2 to properly run on a wider range of environments and dependency versions.

The PSSR2 package comprises six submodules under the main pssr module.

- train: Training and optimization functions
- predict: Prediction and benchmarking functions
- data: Datasets and functions for handling and generating paired high-low-resolution image pairs
- crappifiers: Pre-implemented and user definable crappifiers for semi-synthetically degrading high-resolution images into their low-resolution counterparts
- models: Pre-implemented neural network architectures
- util: Various utility functions

The majority of workflow configuration can be done solely in the selection and definition of objects within these modules. For many use cases, all relevant logic is predefined within corresponding objects, allowing users to remove unnecessary boilerplate code if desired. If accessing PSSR2 via its package interface, the user is not required to write more than a few lines of code defining objects to train and/or run a state-of-the-art model.

Perhaps the greatest source of workflow variance is in the input format and application of microscopy data. To efficiently provide a standardized interface for data loading, we separate our datasets into two categories: image datasets, which deal with preprocessed images, and sliding datasets, which deal with unprocessed data across multiple tiles within a larger image (i.e. a large image to be divided into multiple smaller images). Tiles are defined by horizontal and vertical positions within an image if applicable, where any additional dimensional data is separated into multiple frames of the same tile as set by a user-defined configuration. We separate training and validation sets randomly by tile, rather than by frame, because different frames from the same tile may be overly similar to one another, which may lead to undesirable training data leakage or overfitting. Both dataset types are capable of model training and prediction. In training, datasets automatically generate semi-synthetic low-resolution images from given high-resolution images using a configurable “crappifier”. For model predictions, datasets load only low-resolution images to be restored (i.e. “decrappification”). For model benchmarking, we also provide datasets that omit the crappification of high-resolution images, instead loading real-world high-low-resolution image pairs.

We designed datasets in this manner to be effectively “plug-and-play” while still being comprehensive for as many applications and data types as possible. Additionally, the usage of n-dimensional images is supported, including those with asymmetrical amounts of dimensions between model inputs and outputs, along with the training of models for image denoising tasks. While models could be trained on multidimensional images in the prior PSSR implementation, we expand the scalability and viability of such an approach beyond the single-frame and multi-frame temporal super-resolution approaches previously used.

Various pre-implemented neural network architectures for image super-resolution are accessible from within the package. The included implementations are modified to strip away excess dependencies and bloat, and are standardized to be easily understandable for inexperienced users. However, these are solely for ease of use, and more advanced users are free to use any PyTorch-based neural network architecture.

As the majority of PSSR2 functionality was designed to be accessed through simple object interfaces, it allows for the seamless implementation of said functionality into external applications. In addition to accessing PSSR2 as a Python package, its installation comes included with a Command Line Interface (CLI) and a Napari graphical user interface plugin, each with the same core features as the Python package. Both the CLI and Napari plugin allow training and prediction using custom defined datasets and models. Additionally, the latter allows for runtime monitoring of model output images in multidimensional spaces within the Napari viewer. All functions and objects are fully documented in docstrings visible in interactive programming environments, which are also available along with a user guide in the online documentation.

While all accessibility features of PSSR2 are not mutually exclusive to it, the aforementioned accessibility elements are often not present in other research software. For example, the CSBDeep toolbox for CARE super-resolution/denoising is notable for being an installable software package with documentation. However, some accessibility aspects of CSBDeep are not as developed as in PSSR2, such as its less exhaustive CLI and documentation. Although there is nothing prohibitive of custom user implementation, many features of PSSR2 are also not supported out of the box, such as our improved crappifiers or runtime data generation. In the original manuscript, the previous implementation of PSSR was compared to CARE, where PSSR either met or exceeded the performance of CARE on both single-frame and multi-frame fluorescence images. The performance of PSSR2 exceeds the performance of the previous PSSR, as we discuss later in this manuscript.

### Advancements in PSSR2

PSSR2 overhauls the semi-synthetic data generation process (i.e. “crappification”) of its predecessor, contributing to increased robustness and prediction accuracy on real-world data. In the crappification process of the prior PSSR implementation, semi-synthetic low-quality images (low resolution, high noise) are generated from real-world high-quality images (high resolution, low noise) by adding noise and then downsampling. However, by adding noise first, adjacent noise values are averaged together in downsampling contributing to lower noise variance, especially in value dependent noise distributions such as Poisson noise. Though a seemingly minor change, we found that by adding noise after downsampling, semi-synthetically generated low-resolution training images are more representative of real-world images, thereby enabling more accurate model training. We found that this allowed Poisson noise to be used to great success, where even though it is most statistically similar to the real-world noise processes in a point-scanning system, the prior implementation found it performing poorly.

We also allow training set images to be crappified on statistically independent noise intensity values rather than a set intensity across all images, which allows trained models to function on a wider range of input data qualities. Additionally, by generating training images during runtime rather than before training as did the previous implementation, it allows the same image to receive many distinctly different crappifications (random noise, random intensity) across all training epochs. With both changes in data augmentation combined we can effectively increase the size of the training dataset by a large factor, reducing overfitting and increasing robustness. This improved crappifer methodology is the main driver of increased PSSR2 performance, as it allows for neural network training on data more representative of real-world low-resolution acquisitions.

We also developed a methodology for measuring the amount of noise present in a low-resolution image when compared to its corresponding high-resolution image for use in properly quantifying crappifier intensities for more accurate training data generation. The high-resolution image is first downsampled to create a “noiseless” low-resolution image, the values of which are then subtracted from the true low-resolution image to yield a noise profile, i.e. an n-dimensional array representing the noise in the low-resolution image. Crappifier parameters are then approximated by Bayesian optimization using Gaussian processes [4], minimizing the distribution of noise profile values of the semi-synthetic low-resolution image generated from fit crappifier parameters against that of the true low-resolution image. This methodology allows for the direct quantification of crappifier parameters for a given set of paired high-low-resolution images, which can then be used to generate synthetic low-resolution images of similar quality, without human guesswork of these parameters. It is important to note that this does not take into account the possibility of spatial structure for noise, and assumes spatially independent noise (i.e. noise randomly spread across the image).

## Results

We benchmarked the real-world improvements of PSSR2 using a test dataset consisting of 42 aligned image pairs from different regions of the same tissue sample for both high-resolution (512×512 pixels, 2nm pixel size) and low resolution (128×128 pixels, 8nm pixel size) images. Images were super-resolved using a ResUNet neural network architecture, similar to the one used by PSSR [5]. While the ResUNet architecture used in our benchmarking for PSSR2 has 47 trainable layers versus the 34 trainable layers used in benchmarking PSSR, we found these additional layers themselves contribute very little to increased performance. The model was trained for 20 epochs on a runtime-crappified dataset of 38,880 images acquired by electron microscopy, taking approximately 6 hours to train on a Quadro P6000 GPU. For both use-cases, our benchmarked models were trained with MS-SSIM + L1 loss [6]. We found this loss function provided improved visual clarity over L2 (mean squared error) loss, which was used to train the model in the prior PSSR implementation. Performance is measured as the ability of the model to accurately restore the low-resolution images to their high-resolution counterparts, with a simple bilinear upscaling operation used as a control. These training and testing datasets were acquired from the original PSSR manuscript [1], in which ethics approval for animal work was obtained.

**Fig. 1:**
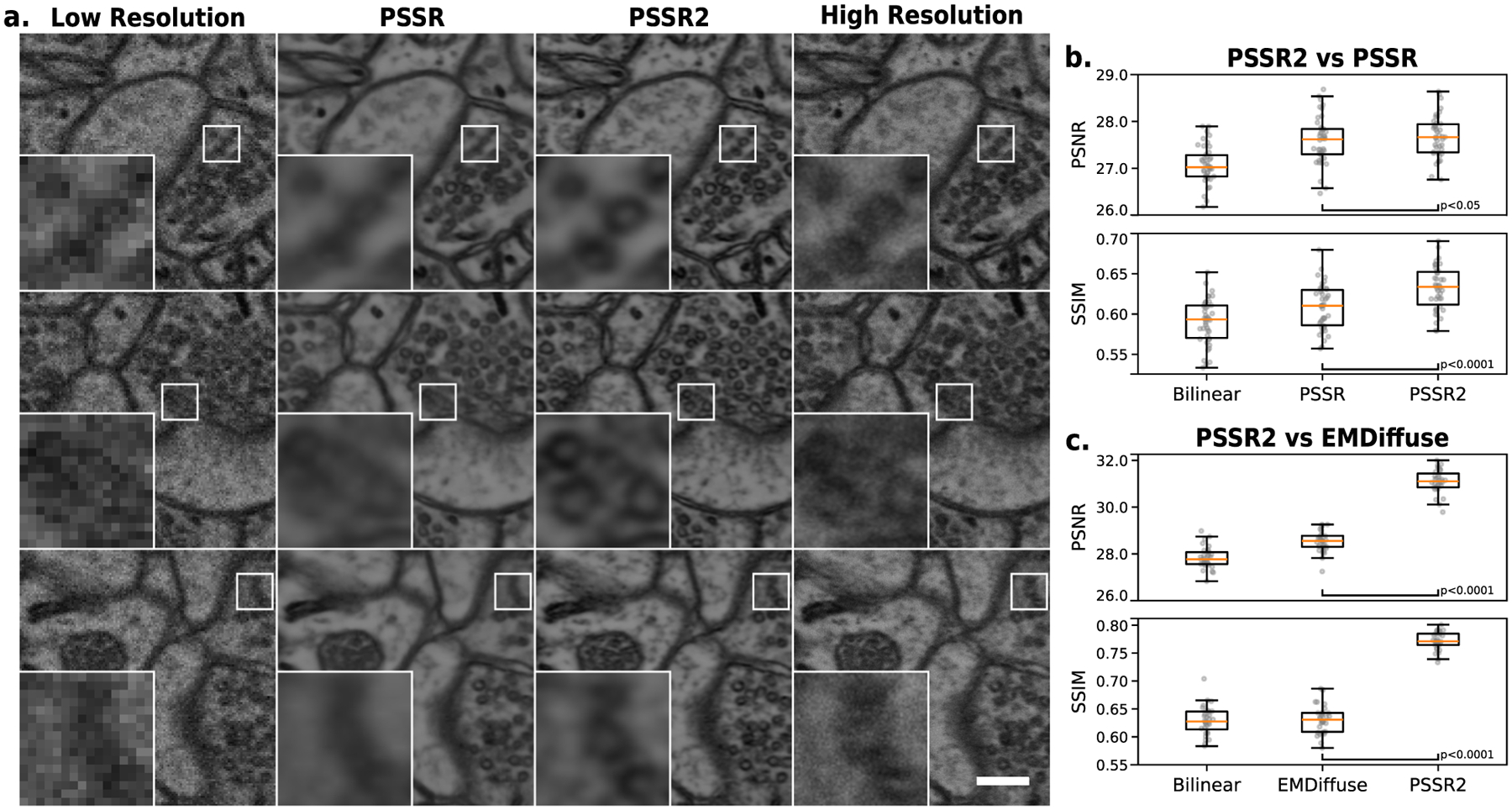
Evaluation of PSSR2 against state-of-the-art microscopy image restoration methods on paired high-low-resolution test sets. **a** Super-resolution ResUNet model predictions of both PSSR2 and the prior PSSR implementation, in comparison to low-resolution inputs and high-resolution ground truth from a test set of 42 paired high-low-resolution images. For each set of corresponding images, a close up shows the same region of interest where PSSR2 predicts cellular structures (e.g., vesicles) more accurately than PSSR. Scale bar is 0.2μm. **b** Image restoration performance comparison of ResUNet super-resolution models using the same test set of 42 images trained with either the prior PSSR implementation or PSSR2, benchmarked against a bilinear upscaling control. **c** Image denoising performance of PSSR2-trained ResUNet model against an EMDiffuse-pretrained model on a separate EMDiffuse test dataset of 32 paired high-low-quality images, with a bilinear upscaling control. All *p*-values measure the significance of metric performance of PSSR2 against a given image restoration method (b, PSSR; c, EMDiffuse) by paired t-test

In our testing, we found that PSSR2 increased model prediction accuracy when compared to the prior PSSR implementation in terms of both PSNR (Peak Signal-to-Noise Ratio) and SSIM (Structural Similarity), widely used metrics for quantitatively measuring the similarity between correlated images (Fig. 1b). PSNR measures the pixel-based error between images, while SSIM measures higher order visual similarities and is more indicative of visually perceived quality. Strikingly, the SSIM performance uplift of PSSR2 against the control more than doubled that of the prior PSSR implementation (130% relative increase, *p* < 0.0001), with a less drastic performance uplift in terms of PSNR (16% relative increase, *p* < 0.05).

To prove the merits of PSSR2 on multiple training tasks, we also benchmarked image denoising against EMDiffuse, another state-of-the-art microscopy image restoration tool utilizing diffusion-based deep learning models [7]. Using the provided pretrained EMDiffuse model on its provided test dataset for 32 images from different regions of the same tissue sample, we found that the image denoising predictive accuracy of PSSR2 surpasses that of EMDiffuse in terms of pixel-based metrics (Fig. 1c), proving its usefulness in a variety of scenarios. The performance uplift of PSSR2 against the control is much higher than that of EMDiffuse in terms of both PSNR (376% relative increase, *p* < 0.0001) and SSIM (0.14 absolute increase, *p* < 0.0001).

## Discussion

We found that PSSR2 achieves state-of-the-art image restoration results on both super-resolution and denoising tasks. In the results of the super-resolution task, PSSR2 achieves a much greater performance uplift in the SSIM metric than in the PSNR metric. This suggests that while PSSR could reliably super-resolve low-resolution images with a relatively low pixel-wise error, the predicted images lacked the higher order visual similarities to real-world images that PSSR2 was able to extract. In context of the properties that each metric measures, these results are indicative of greater visual clarity and more accurate texture in PSSR2 output. This is supported by visual examination of the images generated by both PSSR2 and the prior PSSR implementation from the test set, each using the same low-resolution images as model inputs. The increase in image clarity in the PSSR2 generated images is apparent, with decreased blur and increased sharpness when compared to its predecessor (Fig. 1a). The elimination of these artifacts, while maintaining robust model performance, should improve the performance of any additional or downstream algorithms/workflows.

In the results of the image denoising task, PSSR2 demonstrates increased predictive accuracy when compared to EMDiffuse. In visually examining image predictions from both algorithms, and supported by the test metrics, PSSR2 tends to preserve the position of image elements more often than EMDiffuse, demonstrating the reliability of PSSR2.

PSSR2 models should only be applied to data sufficiently similar to training data for the best accuracy. The provided dataset objects should be sufficient for users to train their own models on custom data according to their needs. It is also strongly encouraged to validate model performance on real-world ground truth data. More specifically, we strongly recommend that the target downstream analyses of PSSR2 output match the accuracy and precision of analyses performed on ground truth data.

## Conclusion

We present here a user-friendly and robust Python package capable of state-of-the-art deep learning-based microscopy super-resolution, easily accessible through its integrated CLI and Napari plugin in addition to a Python interpreter. PSSR2 allows for the efficient acquisition of high quality microscopy images Offering many configurable objects and tools for a variety of use cases while being concise and approachable, PSSR2 is designed to be accessible to most imaging and biology researchers through its simple yet expandable interface. PSSR2 utilizes the widely used PyTorch library, allowing for simple integration with state-of-the-art models and architectures. Importantly, PSSR2 expands on the prior implementation of PSSR to provide more realistic and efficient model training on a wide variety of microscopy data and use-cases. PSSR2 boasts a significant increase in image quality from its predecessor, and outperforms other image restoration approaches in accuracy. We hope this update provides a meaningful step towards democratizing access to image enhancement tools for the biological imaging community.

## Data Availability

PSSR2 is a cross-platform Python package distributed on the Python Package Index. The source code for the CLI and Napari plugin are available under the MIT license at https://github.com/ucsdmanorlab/PSSR2. The original datasets and figures generated and/or analysed during the current study are available in the Zenodo repositories: PSSR2 Data and Figures: https://zenodo.org/records/14060867. EMDiffuse Dataset: https://zenodo.org/records/10205819224.
